# Assessment of Dietary Adequacy and Quality in a Sample of Patients with Crohn’s Disease

**DOI:** 10.3390/nu14245254

**Published:** 2022-12-09

**Authors:** Alexandra Karachaliou, Mary Yannakoulia, Maria Bletsa, Gerassimos J. Mantzaris, Emmanuel Archavlis, George Karampekos, Maria Tzouvala, Giorgos Bamias, George Kokkotis, Meropi D. Kontogianni

**Affiliations:** 1 Department of Nutrition & Dietetics, School of Health Sciences and Education, Harokopio University of Athens, 70 El. Venizelou Avenue, 176 76 Kallithea, Greece; 2 Department of Nutrition and Dietetics, ‘’Sotiria’’ Thoracic Diseases Hospital, 152 Mesogion Avenue, 115 27 Athens, Greece; 3 Department of Gastroenterology, ‘’Evangelismos-Ophthalmiatreion Athinon-Polykliniki’’ General Hospital, 45–47 Ypsilantou Street, 106 76 Athens, Greece; 4 Department of Gastroenterology, General Hospital of Nikaia Piraeus “Agios Panteleimon”-General Hospital Dytikis Attikis “Agia Varvara”, 3 Dim. Mantouvalou Street, 184 54 Athens, Greece; 5 GI Unit, 3rd Academic Department of Internal Medicine, “Sotiria’’ Thoracic Diseases Hospital, Medical School, National and Kapodistrian University of Athens, 152 Mesogion Avenue, 115 27 Athens, Greece

**Keywords:** Crohn’s disease, dietary intake, dietary habits, dietary adequacy, nutrients, food groups, Mediterranean diet, disease activity, under-reporting

## Abstract

Both under-and over-nutrition are prevalent in patients with Crohn’s Disease (CD). The aim of the present study was to evaluate dietary intake and compare it with relevant recommendations during active disease and remission, also taking into consideration the adequacy of energy reporting. Dietary quality was assessed through adherence to the Mediterranean diet and to the European dietary guidelines for cardiovascular disease prevention (CVD-score). Malnutrition was diagnosed with the GLIM criteria. There were 237 patients evaluated (54.9% males, 41.3 ± 14.1 years and 37.6% with active disease). In the total sample, high prevalence of overweight/obesity (61.6%) and low prevalence of malnutrition (11.4%) were observed, whereas 25.5% reported low protein intake in the sub-sample of adequate energy reporters. The mean MedDietScore was 28.0 ± 5.5 and the mean CVD-score was 5.25 ± 1.36, both reflecting moderate dietary quality. Patients with active disease reported higher prevalence of low protein intake, lower carbohydrate, fibers, fruits, vegetables, legumes, and sweets consumption and a lower MedDietScore compared to patients in remission. Consumption of fibers, fruits, vegetables, and legumes while in remission did not result in reaching the recommended intakes, and dietary quality was low as reflected by the MedDietScore. In conclusion, both protein undernutrition and energy overconsumption were prevalent in the current sample and overall patients adhered to a moderate quality diet irrespective of disease stage.

## 1. Introduction

Patients with Crohn’s disease (CD) often experience several nutritional disorders such as malnutrition, sarcopenia, and overweight and obesity, leading to an adverse course of the disease, reduced efficacy of treatment, more frequent complications and hospitalizations, and reduced health-related quality of life [1,2,3,4]. Inadequate dietary intake, malabsorption, inflammation, increased loss of nutrients from the gastrointestinal tract and different pharmacologic interventions, mostly corticosteroids, are the most prevalent factors contributing to the abovementioned nutritional disorders [5,6]. 

Early identification of reduced or/and inadequate energy/nutrient intake remains important for early detection of potential nutritional deficiencies. A recent systematic review with meta-analysis conducted by Lambert et al. [7] including 14 studies that evaluated dietary intake of patients with CD and concluded that they had lower energy and protein intake compared to dietary reference values. Compared with healthy age- and sex-matched controls, patients with CD consumed less protein and fiber [7], whereas on a food group level, patients with CD had lower consumption of bread and cereals, fruits and vegetables, legumes and dairy products than recommended [7]. However, most of the studies included in this review had small sample sizes and several methodological limitations, such as using different ways of assessing dietary intake and disease activity, use of different dietary reference intake values, inclusion of patients mostly in remission and no consideration of potential low energy reporting. It is already known that patients with CD have impaired nutritional status mainly in active disease due to the presence of malabsorption, inflammation and oxidative stress [8]. However, whether nutritional disorders are being overcome during remission and whether patients’ dietary intake is more balanced and healthy remains understudied during this phase [9]. Limited data also support unbalanced dietary intakes during remission mainly because of fear concerning the consumption of fiber-rich foods, misconceptions regarding the recommended diet or even wrong guidance from health care professionals, peers or the internet [10,11,12]. 

Hence, the aim of the present study was to evaluate dietary intake in terms of quantity and quality and to compare them with relevant recommendations during both active disease and remission in a sample of patients with CD, also taking into consideration the adequacy of energy reporting. 

## 2. Materials and Methods

### 2.1. Study Sample

**Figure 1:**
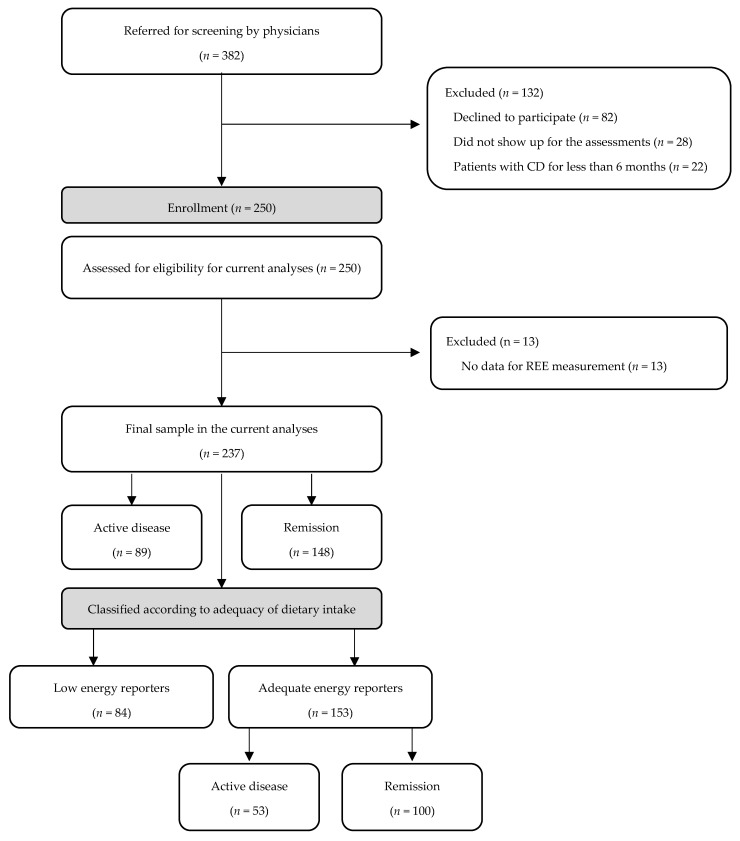
CONSORT chart. From November 2018 to November 2019, 382 patients with CD were referred by collaborating physicians for screening. Of the 382 patients, 82 declined to participate, 28 did not show up for the assessments and 22 were diagnosed with CD for less than 6 months. In total, 250 patients with CD included in a cross-sectional evaluation of nutritional status, were assessed for eligibility in the current analyses. Thirteen patients were excluded from the present analyses, because of missing data regarding REE measurement, leading to a final sample of 237 patients. Of the final sample, 89 patients were in active disease and 148 in remission. Four patients were on a weight loss diet and the rest were further classified according to adequacy of dietary intake based on Goldberg et al. criteria to 80 low energy reporters and 153 adequate energy reporters. Among the adequate energy reporters, 53 had active disease and 100 were in remission. Abbreviations: CD: Crohn’s Disease, REE: Resting Energy Expenditure.

In this cross-sectional study, patients with confirmed CD for more than six months were included from the three following outpatient gastroenterology clinics: Department of Gastroenterology, General Hospital of Athens “Evangelismos-Ophthalmiatreio Athinon-Polykliniki”, Department of Gastroenterology, General Hospital of Nikaia Piraeus “Agios Panteleimon”, General Hospital Dytikis Attikis “Agia Varvara” and GI-Unit, 3rd Academic Department of Internal Medicine, “Sotiria” Hospital of Athens. Inclusion and exclusion criteria and diagnosis procedure are described in detail elsewhere [13]. Patients with CD for less than 6 months were excluded from the study, in order to include only patients with more stable CD and dietary habits. For current analyses, 13 patients with missing data regarding resting energy expenditure (REE) measurement were excluded from the initial sample of 250 patients, leading to a final sample of 237 participants (Figure 1). All participants signed a written consent form. The study protocol was approved by the Bioethics Committee of Harokopio University, the Scientific Committees of the three participating hospitals and was registered in ClinicalTrials.gov (NCT03871634).

### 2.2. Medical Assessment

During the medical assessments, physicians asked patients about their education level, work and marital status as well as other diseases and medication they used. Montreal classification was used to categorize patients according to age at diagnosis (<16, 17–40, >40 years), disease location (ileal, colonic, ileocolonic) and behavior (non-stricturing/non-penetrating, stricturing, penetrating) [14]. Disease activity was assessed endoscopically using the Harvey-Bradshaw Index (HBI) [15]. Based on a treat-to-target approach [16], patients with endoscopic remission defined as resolution of ulceration at ileocolonosopy, clinical remission (HBI < 5) and normal CRP levels were categorized as “in remission.” In the absence of recent endoscopy, clinical remission (HBI < 5) and normal levels of CRP were necessary to categorize a patient as “in remission.” In the event of cases with high CRP levels but with HBI < 5, patients’ general conditions were assessed to exclude other potential causes of increased CRP (e.g., infections, dental problems etc.) [16,17]. 

### 2.3. Anthropometry, Body Composition and Assessment of Malnutrition and Sarcopenia

Body weight and height were measured (Seca Alpha, Model 770, Hamburg, Germany) and body mass index (BMI) was calculated according to WHO guidelines [18]. Moreover, desired body weight was calculated using body weight corresponding to BMI 20 kg/m^2^ for patients with BMI < 18.5 kg/m^2^, actual body weight for patients with BMI 18.5–24.9 kg/m^2^ and BMI corresponding to 25 kg/m^2^ for patients with BMI > 25 kg/m^2^ [19,20,21]. Patients were asked about changes in their body weight or whether they had followed a weight loss diet over the past two weeks.

Body composition analysis was performed using the Dual X-ray Absorptiometry (DXA) method (Lunar DPX-MD, Lunar Corp., Madison, WI, USA) and the Appendicular Skeletal Mass Index (ASMI) was calculated. Low ASMI was defined as <7 kg/m^2^ for men and <5.4 kg/m^2^ for women [22]. Muscle strength was assessed using handgrip strength with cut-off values of <27 kg for men and <16 kg for women [22]. Sarcopenia was defined using European Working Group on Sarcopenia in Older People (EWGSOP2) criteria as low muscle strength and low muscle mass [22]. GLIM (Global Leadership Initiative on Malnutrition) criteria [23] were used to assess the presence of malnutrition. Malnutrition, according to GLIM criteria, was diagnosed if a patient had at least one phenotypic (low BMI, nonvolitional weight loss, reduced muscle mass) and one etiologic criterion (reduced food intake or assimilation, disease burden/inflammation). Low BMI was defined as <20 kg/m^2^ for participants <70 years and <22 kg/m^2^ for participants >70 years, nonvolitional weight loss was defined as >5% within past 6 months or >10% beyond 6 months and reduced muscle mass was assessed calculating ASMI from DXA and defined as mentioned above. Regarding etiologic criteria, reduced food intake was present if ≤50% of energy requirement for >1 week or any reduction of food intake for >2 weeks was recorded, reduced assimilation was scored in case of extensive active ileal or ileocolonic Crohn’s disease, and inflammation was assessed using CRP levels (CRP > 5 mg/L) or presence of endoscopic inflammation.

### 2.4. Energy Expenditure

Resting energy expenditure (REE) was measured via indirect calorimetry (Ultima™ Series PFX^®^ with gas chromatography, MedGraphics Cardiorespiratory Diagnostics Corporation, St. Paul, MN, USA) for about 20–30 min with a methodology described in detail elsewhere [13]. REE was calculated using the Weir formula [24], without using protein oxidation [25]. Physical activity level (PAL) was assessed using the Athens Physical Activity Questionnaire (APAQ) that was validated for the Greek population [26,27]. Moreover, total energy expenditure (TEE) was calculated multiplying measured REE from indirect calorimetry by PAL. 

### 2.5. Dietary Intake Assessment

Energy and nutrient intake was assessed through two non-consecutive 24-h recalls (one weekday and one weekend day) and a food frequency questionnaire (FFQ) by an experienced trained dietician. 24-h recalls interview was based on the “five-step multiple pass” method proposed by the USDA [28]. Recalls were analyzed using Nutritionist Pro software (2007, Axxya Systems, TX, USA) in terms of energy and macronutrient intake. Nutritional analyses of traditional Greek foods and recipes [29] and nutritional information on oral nutritional supplements (ONS) were also included in Nutritionist Pro database. 

Goldberg et al. [30] cut-off points were used for the assessment of low energy reporting. Low energy reporting was defined as ratio of energy intake to TEE less than 0.98, while a ratio ≥0.98 indicated adequate energy reporting. Energy (as kcal/day and kcal/kg actual and desired body weight/day) was compared to the actual needs as calculated by TEE. Normal-weight patients were categorized as having adequate energy intake if it was within ±5% of TEE, higher if it was ≥105% of TEE and lower than their needs when it was ≤95% of TEE. Furthermore, protein intake (as g/day and g/kg actual and desired body weight/day) was compared to the ESPEN guidelines [31]. Patients were categorized as having adequate protein intake when their protein intake was 1.2–1.5 g/kg body weight/day for active disease and 1 g/kg body weight/day for remission based on ESPEN’s guidelines, lower protein intake when their protein intake was <1.2 g/kg body weight/day for active disease and <1 g/kg body weight/day for remission and higher protein intake when they exceeded the thresholds from ESPEN’s guidelines [31]. The intake of other macronutrient (carbohydrates, fatty acids, etc.) was compared with EFSA’s recommendations for healthy adults [32]. 

The FFQ was used to assess dietary habits and food group consumption during the last month before the assessment [33,34]. Food groups and portion sizes are presented in Table S1 [29,35,36,37]. Food group consumption was compared with national dietary guidelines for the adult Greek population [38]. Adherence to the Mediterranean diet was assessed using the MedDietScore [33,34], which is based on the consumption of foods from nine food groups, olive oil and alcoholic beverages. Values range from 0 to 55, with higher values indicating higher adherence to the Mediterranean diet [33,34]. Moreover, based on recent European dietary guidelines for cardiovascular disease (CVD) prevention, a relevant score was calculated to estimate adherence to the European Society of Cardiology (ESC) recommendations (excluding recommendation for salt intake that could not be calculated in the present study). Participants received a score of 1 for each of the recommendations they reached the aim, otherwise they received a score of zero. After adding the scores for the ESC recommendations, a final score was calculated ranging from 0 to 11, with higher values indicating higher adherence to these recommendations [39]. 

### 2.6. Statistical Analysis

These are secondary analyses of a cross-sectional study aiming to estimate the prevalence of malnutrition in patients with CD. Statistical analyses were performed using IBM SPSS version 23 (IBM Corp. Released 2016. IBM SPSS Statistics for Windows, Version 24.0. Armonk, NY, USA: IBM Corp.) and STATA version 15 (M. Psarros & assoc. Sparta, Greece). The level of statistical significance was set at 0.05. Shapiro-Wilk test and Q-Q plots were used to test the normality of continuous variables. Categorical variables were presented as relative and absolute frequencies, continuous normally distributed variables as mean ± standard deviation (SD) and skewed variables as median and 25th and 75th percentiles. Differences between active disease and remission were evaluated using independent t-test or Mann-Whitney rank tests for normally and skewed continuous variables, respectively, and chi-square tests were used for comparisons between categorical variables. Tukey test or multiple Mann-Whitney rank tests were used in post-hoc analyses to identify groups which differed significantly. Paired-sample t-test and Wilcoxon signed-rank test were used to compare energy intake and TEE for normally distributed and skewed continuous variables, respectively.

## 3. Results

### 3.1. Descriptive Characteristics and Differences between Low and Adequate Energy Reporters

Two hundred thirty-seven patients (130 males) aged 41.3 ± 14.1 years and with mean BMI 27.3 ± 6.0 kg/m^2^ were included in the analyses. Most of the patients had ileal (46.8%) or ileocolonic disease (43.3%) and only 11.4% of them had isolated upper gastrointestinal disease. Patients’ characteristics in the total sample and according to disease activity are presented in Table 1. A high prevalence of overweight/obesity and a low prevalence of malnutrition were reported in the total sample. Prevalence of sarcopenia was very low (2.2%) in the total sample and did not differ between active disease and remission. Patients with active disease had higher HBI [4.0 (2.0, 6.0) vs. 1.0 (0.0, 2.0), *p* < 0.001] and higher prevalence of malnutrition (23.5% vs. 4.8%, *p* < 0.001) compared with those in remission (Table 1). Four participants were on a weight loss diet, while 80 (33.8%) were categorized as low energy reporters (LERs). LERs were mainly females (*p* = 0.030), with more than 12 years of education (*p* = 0.003) and had higher BMI (29.7 ± 6.5 kg/m^2^ vs. 26.0 ± 5.4 kg/m^2^, *p* < 0.001) and REE (1912 ± 450 kcal/day vs. 1665 ± 401 kcal/day, *p* < 0.001) compared to adequate energy reporters (AERs). LERs and AERs did not differ in terms of HBI, location of disease, ASMI, handgrip strength, presence of sarcopenia and malnutrition, medication and level of physical activity (data not shown). A comparison of energy, macronutrient intake and food group consumption between LERs and AERs are presented in Tables S2 and S3.

### 3.2. Energy and Macronutrients’ Intake 

Table 2 presents energy and macronutrient intakes for the total sample and for AERs after also excluding those on a weight loss diet and according to disease activity. Given the high prevalence of overweight and obesity, energy intake was expressed per kg desired body weight. In the total sample, median energy intake was 28.2 (22.8, 37.4) kcal/kg desired body weight/day, which did not differ between disease-activity groups, whereas high energy intakes were reported in the AER subgroup [34.2 (28.0, 41.6) kcal/kg desired body weight/day], as anticipated given the high prevalence of overweight/obesity. In the sub-group of patients of normal-weight (*n* = 84), 45.2% reported lower energy intake than needed and this percentage was 27.4% when only AERs were included (*n* = 62). 

Regarding macronutrient intake, 40.5% of patients in the total sample reported lower protein intake than recommended based on ESPEN guidelines, with this percentage being 62.9% in those with active disease and 27.1% in those in remission (*p* < 0.001). Patients with lower protein intake than recommended were mainly females (54.5% vs. 39%, *p* = 0.02), of older age (45.1 ± 14.7 years vs. 38.7 ± 13.2 years, *p* < 0.001), with higher BMI (30.3 ± 6.5 kg/m^2^ vs. 25.1 ± 4.7 kg/m^2^, *p* < 0.001) and lower PAL (1.29 ± 0.19 vs. 1.35 ± 0.25, *p* = 0.04) compared with those with protein intake within recommendations (data not shown). When protein intake was expressed as a percentage of TEI, patients with active disease reported higher intake compared with those in remission (19.1 ± 5.0% vs. 17.6 ± 4.3%, *p* = 0.01), whereas differences were not significant when expressed as grams per kg actual or desired body weight (Table 2). From the reported protein intake, 65.3% was animal protein (from dairies, eggs, meat, poultry and fish) and a high percentage of animal protein was recorded both in those with active disease and in remission. Carbohydrate intake was lower than that recommended by EFSA with a rather high intake of sugars (exceeding the threshold of 10% as suggested by WHO [40]), total fat intake was higher than what is recommended for the general population and saturated fat (SFA) intake was higher than the recommended goal of <10% for CVD prevention [39]. Patients in remission reported higher carbohydrate (*p* = 0.01) and fiber intake (*p* < 0.001) than those with active disease, however still lower than the recommended (Table 2). 

Similar analyses of dietary intake were repeated only in the sub-sample of the AERs. Of AERs, 25.5% reported lower protein intake than what is recommended based on ESPEN guidelines with most of them having active disease (47.1% vs. 14.0%, *p* < 0.001). Similar results with the total sample were observed for AERs regarding macronutrient intake, except for protein intake where no statistically significant differences between active disease and remission were observed (Table 2). An increased percentage of animal protein intake (64.1%) was recorded also in AERs. 

### 3.3. Food Groups Consumption and Adherence to Mediterranean Diet and European Dietary Guidelines for CVD Prevention

Regarding food group consumption when compared to Greek dietary guidelines, patients in the total sample reported lower consumption of dairy products, non-refined cereals, fruits and vegetables, legumes and fish and higher consumption of refined cereals, red meat and sweets (Table 3). The MedDietScore of the total sample was 28.0 ± 5.5, reflecting a moderate adherence to the Mediterranean diet, and total diet score for primary CVD prevention was 5.25 ± 1.36, reflecting a moderate adherence to the recently published European guidelines for CVD prevention [39]. According to disease activity, patients with active disease reported lower consumption of fruits and vegetables (*p* = 0.01), legumes (*p* < 0.001) and sweets (*p* = 0.05) and lower MedDietScore (*p* = 0.01) compared to patients in remission (Table 3). Although patients in remission reported higher consumption of fruits, vegetables and legumes, they did not reach the recommended intakes and dietary quality remained moderate as reflected by the MedDietScore. Similar results have also been observed for AERs, except for non-refined cereals (with those in remission reporting higher intakes, although still too low), sweets (with patients in both disease-activity groups equally consuming high amounts of sweets) and total score for CVD prevention (which was higher in those in remission) (Table 3).

## 4. Discussion

The aim of the present study was to assess energy and macronutrient intake, food group consumption and dietary quality in a sample of patients with CD and to explore whether dietary intake is optimized during remission when disease symptoms are partly alleviated. A double burden of malnutrition (both protein undernutrition and energy overconsumption/high prevalence of overweight and obesity) was prevalent in the present sample. Specifically, 61.6% were overweight/obese, whereas only 11.4% were malnourished based on the GLIM criteria in the total sample, however around 25% reported lower protein intake than that recommended by ESPEN’s guidelines in the subgroup of AERs. Those with active disease reported higher prevalence of low protein intake and lower intakes of carbohydrates, fibers, fruits, vegetables, legumes, sweets, as well as lower adherence to the Mediterranean diet compared to those in remission. Interestingly, in the group of patients in remission, although higher intake of fruits, vegetable and legume consumptions were reported, they did not reach the recommended intakes and dietary quality remained moderate as reflected by the MedDietScore and CVD prevention score.

Energy and protein intake within recommendations are important for optimizing nutritional status and outcomes in patients with CD [41]. Previous studies exploring habitual dietary intake in patients with CD have reported conflicting results. A recent systematic review with meta-analysis [7] evaluating 19 studies and exploring dietary intake of adults with inflammatory bowel disease concluded that patients with CD had lower energy intake at about 300–400 kcal than recommended and met or exceeded recommendations for protein intake. However, most of the studies included in this review used different dietary reference values for energy and protein intake and included mainly patients in remission, and under-, normal- or slightly over-weight patients (17.4–27.4 kg/m^2^). The present study used the most recent dietary guidelines for patients with CD to assess adequacy of dietary intake and included also around 38% of patients with active disease, trying for a better reflection of dietary intake in both disease-activity groups compared to other studies including mainly patients in remission or a small number of patients with active disease. Nevertheless, results of the present study revealed a combination of high prevalence of overweight and obesity (almost 62%) with low prevalence of malnutrition (almost 11%), also confirmed by other studies from the last decade reporting a high prevalence of overweight and obesity (40–55%) in patients with CD [42,43]. This finding leads to the conclusion that at least in Greece where prevalence of overweight and obesity is high in the general population [44], this problem also remains prevalent in patients with CD and therefore contributing probably to an increased risk of chronic diseases and long-term comorbidities [42,43]. Even in the sub-group of normal-weight participants, those that reported lower than the required energy intake had a rather low median energy deficit of around 150 kcal (data not shown), without any differences between active disease and remission. 

Among AERs, one out of four patients recorded lower protein intake than what is recommended by ESPEN’s guidelines, and were mainly patients with active disease, females, older in age, with higher BMI and lower PAL. Nevertheless, both in the total sample and in those with low protein intake, around 65% of the protein was from animal sources (dairies, eggs, meat, poultry and fish) providing proteins of high biological value. Therefore, the clinical implication of low protein intake should be interpreted with caution, given the fact that current recommendations for protein intakes are not based on robust data and are rated as, “Good practice points. Recommended best practice based on the clinical experience of the guideline development group” in the ESPEN’s document. Hence, well-designed studies are required to examine whether these recommendations reflect patients’ actual needs or should also be revised given the low prevalence of sarcopenia and malnutrition as diagnosed by valid diagnostic algorithms. However, until more data becomes available, efforts should focus on early identification of patients with low protein intakes even in patients with adequate or more than adequate energy intake, and intervention towards optimization of protein intake, throughout the disease course, remains a relevant aim of nutrition care plans in patients with CD.

Regarding other aspects of dietary intake, in the present sample patients’ total carbohydrate intake was lower than the recommended and was accompanied by high sugar and low fiber intakes. As expected, those with active disease reported lower fiber intake as reflected also by lower consumption of fruits, vegetables and legumes compared with patients in remission. Presence of active CD is anticipated to lead to a reduction of dietary intake due to anorexia, inflammation and symptoms like abdominal pain, distention and/or diarrhea, especially through restriction of foods containing lactose or fibers [5]. A recent systematic review with meta-analysis [7] concluded that patients with CD had lower fiber intake than what is recommended, with those with active disease reporting slighter lower fiber intakes compared to those in remission, while no data were available for sugar intake in patients with CD in both disease-activity groups. Additionally, in the present study, although patients in remission reported higher intakes of fiber and related food groups compared to those with active disease, consumption was low compared to the desired. Specifically, they reported an increased consumption of sweets and a reduced consumption of fruits, vegetables, legumes, non-refined cereals and dairy products compared to recommendations, which could lead to adverse effects on patients’ overall health. Both the British Society of Gastroenterology guidelines [45] and the ESPEN’s guidelines for patients with inflammatory bowel disease [46] conclude that there is no specific diet to be followed during remission and patients are recommended to follow guidelines for the general population, except for those with stricturing disease or food intolerances. Current findings are in line with a recent systematic review describing dietary intake of adults with inflammatory bowel disease [7]. Fiber intake was lower than the recommended values of 25–30 g [7] and patients reported a lower consumption of fruits, vegetables, legumes and dairy products compared to healthy volunteers [7]. Other available studies support a reduced daily fiber intake ranging from 6.13–11.1 g/1000 kcal [47,48,49,50]. It seems that the low fiber diet prescription which is very common during active disease for improving patients’ symptoms, is many times extended also during remission, for fear that their consumption may exacerbate symptoms [45,51]. Adherence to a low fiber diet is only recommended to those patients with active disease and severe flares and those with strictures or obstructive disease [45,51]. In the present study, fiber intake during remission remained low even when those with stricturing disease (*n* = 71) were excluded [median intake 15.2 (11.1, 21.5) g/day]. Unjustified adherence to a low fiber diet during remission often incites fear of triggering symptoms or a new flare-up, and signs the lack of routine follow-up of these patients by a nutrition care team, which is important for the prevention of nutritional deficits, the maintenance of good nutritional status and a balance of gut microbiome throughout the disease course. 

Low consumption of dairy products as well as different sources of fiber have been suggested to favor conditions of gut dysbiosis [52] and undermine gut microbiome composition [52]. Reducing dairy product consumption affects gut diversity, influences the growth of *Bifidobacterium longum subsp. longum* and *Parabacteroides distasonis*, inhibits the growth of *Clostridium perfingers* and *Escherichia coli* [53] and reduces intestinal permeability [54]. Furthermore, a low fiber diet has been related to an increase in IL-6 and TNF-a levels, resulting in mucosal inflammation [55,56] and a reduced production of short chain fatty acids-producing bacteria [57,58,59], whereas a high fiber diet has been related to healthy gut microbiomes with high diversity and reduced CD symptoms [60,61,62]. Moreover, increased SFA intakes can lead to an increased number of sulphate-reducing bacteria, enhancing the risk of developing intestinal inflammation [63] and reducing the overall diversity of the microbiota, including *Bifidobacterium* [64]. In the present study, the combination of high SFA intakes mainly from red meat, low intake of fiber from all food sources and low dairy products consumption implies that adherence to a more westernized dietary pattern could synergistically undermine gut microbiome composition and enhance the induction of intestinal inflammation and long-term related comorbidities [65].

Adherence to a dietary pattern with a variety of fruits, vegetables, non-refined cereals, legumes, nuts and a reduction of animal fat, processed meats and sugars, similar to a Mediterranean-type dietary pattern, is highly recommended by both the British Society of Gastroenterology guidelines and the ESPEN’s guidelines for patients with inflammatory bowel disease without obstructive disease and during attenuation of disease flare-ups [45,46]. For this reason, the present study examined adherence to the Mediterranean diet given that it is also the pattern promoted by the national guidelines. The mean MedDietScore during remission was 28.7 ± 5.5, reflecting a moderate adherence, similar to that previously reported for the general Greek population [66]. Adherence during active disease was, as expected, lower. Adherence to the Mediterranean pattern has also been explored in studies from Australia evaluating 100 outpatient participants with active IBD and in remission, and assessing adherence to the Mediterranean diet using a score outlined from the Australian Guide to Healthy Eating [67] and in Canada in a sample of 67 patients with CD in remission using the PREDIMED criteria [47]. Both studies reported low adherence to this pattern, which may have also been affected by the non-Mediterranean origin of the patients studied. Unfortunately, numerous studies conducted during the last decades in several age groups have shown an abandonment of the Mediterranean diet within the countries of the Mediterranean basin. This finding was also confirmed in the present study of patients with CD, as reflected by the MedDietScore and patients’ reluctance to consume food groups such as fruits, vegetables and legumes together with a high red meat intake, even in the remission phase [68,69]. Therefore, nutrition education and counseling programs should be implemented even in the Mediterranean countries to encourage enhancement of adherence to the Mediterranean diet according to current guidelines for patients with CD. Furthermore, given the fact that patients with CD have increased risk for CVD due to a high inflammatory milieu, both during active disease and in remission phases, when low-grade chronic inflammation has been observed [70], adherence to the recent dietary guidelines for primary CVD prevention was also evaluated. Based on the results, patients reported moderate adherence to these guidelines. To our knowledge, this is the first attempt at exploring adherence to these dietary guidelines; therefore, these results cannot be compared with other results. In total, both dietary indexes calculated in the present study reflect a moderate-quality diet, which when combined with increased inflammation and the increased prevalence of overweight and obesity found in the present sample (almost 62%), should raise awareness about the increased risk for CVD in this population. 

The prevalence of low-energy reporting was rather low in the present sample and sensitivity analyses, and according to the adequacy of energy reporting, did not reveal important differences in dietary intake in terms of the most macronutrients and food groups, or adherence to Mediterranean diet except for the anticipated difference in energy intake that confirmed the high energy intakes of the current sample and in protein intake. Specifically, intake was higher in the AERs sub-sample protein compared to the total sample, leading to a reduced prevalence of low protein intake that should be taken into consideration. In addition, when comparing the two disease-activity groups among AERs, a higher intake of non-refined cereals was reported in remission (although still too low), and an equally increased consumption of sweets in both disease-activity groups, as well as a higher total score for CVD prevention for those in remission. To the best of our knowledge, this is the first study considering the impact of low-energy reporting of dietary intake in patients with CD. Nevertheless, according to the present findings, these results did not present significant differences from that of the total sample on a food group level, which suggests a rather minimal impact of energy reporting to such dietary intake data. However, energy and protein intake were significantly affected which should not be overlooked when assessing adequacy of energy or protein intake. 

The present study has both strengths and limitations. To our knowledge, this is the first study assessing dietary intake in a holistic way including macronutrients and food group consumption, adherence to the Mediterranean diet and to the new recommendations for CVD primary prevention in patients with CD both in active disease and remission, after also taking into consideration dietary under-reporting. In addition, REE was measured using indirect calorimetry and was not calculated using predictive equations. This study included only patients with CD with different disease location and disease activity to allow generalizability of our findings throughout the course of CD. Regarding limitations, a FFQ was used in order to examine food groups consumption in the last month before the assessment, thus patients with active disease may have reported different food groups consumption and not what was actually consumed during the disease flare-ups. Moreover, the 24-h recall method is based on a patient’s memory which involves possibly making errors when recalling details regarding food consumed the previous day. However, using the “five-step multiple-pass” method by an experienced dietician and further exclusion of LERs could minimize this error. 

## 5. Conclusions

High prevalence of overweight/obesity and low prevalence of malnutrition and sarcopenia were observed in the current sample that were, nevertheless, combined with a rather high prevalence of protein intake lower than what is recommended, and with a low quality diet overall as reflected by higher intakes of sugars and SFA, higher consumption of sweets and lower consumption of fruits, vegetables, legumes, non-refined cereals and dairy products. In addition, moderate adherence to the Mediterranean diet and to the CVD primary prevention dietary guidelines were reported, which together with the high rates of overweight, set this population on a high CVD risk. Disease activity was a parameter affecting dietary intake, with those with active disease reporting higher prevalence of protein intake lower than what is recommended by ESPEN’s guidelines, lower carbohydrate and fiber intakes, lower consumption of fruits, vegetables, legumes and sweets and lower adherence to the Mediterranean diet compared to those in remission. However, even if dietary intake was slightly improved during remission, it remained far from what is recommended. Current findings underline the importance and need for a routine assessment of dietary intake throughout the disease course, and for dietary interventions that will improve weight status and dietary intake towards an adequate and high-quality, healthy eating pattern in an effort to optimize disease outcomes and prevent comorbidities.

## Figures and Tables

**Table 1:** Descriptive characteristics of 237 patients with Crohn’s disease and according to disease activity.

	Total Sample (*n* = 237)	Active Disease (*n* = 89)	Remission (*n* = 148)	*p*
**Age (years)**	41.3 ± 14.1	42.2 ± 14.7	40.8 ± 13.7	0.52
**Sex**				0.01
Male	130 (54.9)	55 (61.8)	75 (50.7)	
**BMI (kg/m^2^)**	27.3 ± 6.0	27.6 ± 6.4	27.1 ± 5.8	0.84
**BMI categories (*n*, %)**				0.80
<18.5 kg/m^2^	7 (3.0)	2 (2.2)	5 (3.3)	
18.5–24.9 kg/m^2^	84 (35.4)	34 (38.2)	50 (33.8)	
25.0–29.9 kg/m^2^	82 (34.6)	28 (31.5)	54 (36.5)	
≥30.0 kg/m^2^	64 (27.0)	25 (28.1)	39 (26.4)	
**Educational level (*n*, %)**				0.19
<6 years	23 (9.7)	11 (12.4)	12 (8.1)	
6–12 years	94 (39.7)	39 (43.8)	55 (37.2)	
>12 years	120 (50.6)	39 (43.8)	81 (54.7)	
**Work status (*n*, %)**				0.88
Full time job	150 (63.3)	56 (62.9)	94 (63.5)	
Part time job	21 (8.9)	7 (7.9)	14 (9.5)	
Unemployment	66 (27.8)	26 (29.2)	40 (27.0)	
**Family status (*n*, %)**				0.28
Married	115 (48.5)	42 (47.2)	73 (49.4)	
Unmarried	106 (44.7)	38 (42.7)	68 (45.9)	
Widowed/divorced	16 (6.8)	9 (10.1)	7 (4.7)	
**Disease duration (years)**	7.0 (3.0–13.2)	6.0 (2.0, 14.0)	8.0 (3.0, 13.0)	0.24
**Age at diagnosis (*n*, %)**				0.02
<16 years, A1	22 (9.3)	2 (2.2)	20 (13.5)	
17–40 years, A2	151 (63.7)	62 (69.7)	89 (60.1)	
>40 years, A3	64 (27.0)	25 (28.1)	39 (26.4)	
**Disease location (*n*, %)**				0.10
Ileal, L1	111 (46.8)	44 (50.0)	67 (44.8)	
Colonic, L2	23 (9.9)	4 (4.5)	19 (13.1)	
Ileocolonic, L3	103 (43.3)	41 (45.5)	62 (42.1)	
**Isolated upper GI disease, L4 (*n*, %)**	27 (11.4)	11 (12.4)	16 (10.8)	0.72
**Disease behavior (*n*, %)**				0.28
Non-stricturing, non-penetrating, B1	134 (56.6)	46 (51.7)	88 (59.2)	
Stricturing, B2	71 (30.0)	32 (36.0)	39 (26.5)	
Penetrating, B3	22 (9.2)	6 (6.7)	16 (10.9)	
B2 + B3	10 (4.2)	5 (5.6)	5 (3.4)	
**Perianal disease, *p* (*n*, %)**	33 (14.0)	12 (13.5)	21 (14.3)	0.86
**HBI**	1.0 (1.0–4.0)	4.0 (2.0, 6.0)	1.0 (0.0, 2.0)	<0.001
**Presence of malnutrition (*n*, %)**	27 (11.4)	20 (23.5)	7 (4.8)	<0.001
**Presence of sarcopenia (*n*, %)**	5 (2.2)	3 (3.5)	2 (1.4)	0.29

BMI: Body mass index, GI: Gastrointestinal disease, HBI: Harvey-Bradshaw index, PAL: Physical activity level, REE: Resting energy expenditure. Fat-free mass and fat mass were measured using dual-energy X-ray absorptiometry. Data for continuous variables are presented as means ± standard deviation or median and 25th–75th percentiles and for categorical variables as absolute numbers and relative frequencies. *p*-values were calculated using independent t-test or Mann-Whitney rank tests for normally and non-normally distributed continuous variables, respectively, and chi-square tests for categorical variables.

**Table 2:** Energy and macronutrients intake for 237 patients with Crohn’s disease in the total sample and in adequate energy reporters not on a weight loss diet and according to disease activity.

	Guidelines	Total Sample (*n* = 237, 100%)	Active Disease (*n* = 89)	Remission (*n* = 148)	*p* *	Adequate Energy Reporters (AERs) and Not following a Weight Loss Diet (Ν = 153, 64.6%)	Active Disease (*n* = 53)	Remission (*n* = 100)	*p* **
**Total energy intake (TEI) (kcal/day)**		1933 (1556, 2559)	1921 (1377, 2437)	1966 (1590, 2596)	0.27	2295 (1879, 2899)	2369 (1920, 2983)	2280 (1871, 2884)	0.79
Per kg actual BW (kcal/kg/day)		26.2 (19.2, 34.7)	24.4 (18.2, 32.6)	26.9 (20.0, 35.7)	0.13	31.6 (25.8, 39.1)	30.2 (24.3, 39.3)	31.6 (26.5, 38.7)	0.54
Per kg desired BW (kcal/kg/day)		28.2 (22.8, 37.4)	27.0 (21.7, 37.7)	28.7 (23.9, 37.1)	0.17	34.2 (28.0, 41.6)	33.2 (27.9, 41.4)	34.3 (28.2, 41.6)	0.97
**Protein intake (% TEI)**		18.2 ± 4.6	19.1 ± 5.0	17.6 ± 4.3	0.01	17.5 ± 4.0	17.8 ± 3.8	17.3 ± 4.1	0.47
Animal protein (%)		65.3	67.4	64.0	0.02	64.1	64.9	63.8	0.37
Plant protein (%)		34.7	32.6	36.0	0.63	35.9	35.1	36.2	0.85
Per kg actual BW (g/kg/day)	ESPEN: Active disease: 1.2–1.5 g/kg BW Remission: 1 g/kg BW	1.14 (0.85, 1.46)	1.07 (0.83, 1.57)	1.15 (0.91, 1.44)	0.63	1.36 (1.05, 1.78)	1.30 (0.96, 1.94)	1.37 (1.09, 1.68)	0.89
Per kg desired BW (g/kg/day)		1.26 (1.03, 1.63)	1.22 (1.01, 1.76)	1.28 (1.04, 1.60)	0.91	1.44 (1.17, 1.85)	1.52 (1.10, 1.94)	1.43 (1.18, 1.76)	0.53
Lower than recommended (*n*, %)		96 (40.5)	56 (62.9)	40 (27.1)	<0.001	39 (25.5)	25 (47.1)	14 (14.0)	<0.001
**Carbohydrates (% TEI)**	EFSA: 45–60%	41.2 ± 8.5	39.5 ± 8.6	42.3 ± 8.3	0.01	41.4 ± 8.7	39.4 ± 8.0	42.3 ± 8.9	0.05
**Sugars (%TEI)**	EFSA: As low as possible	12.8 (9.2, 16.3)	12.0 (8.9, 15.6)	13.1 (9.3, 16.4)	0.29	11.7 (8.8, 15.5)	10.7 (7.5, 15.0)	12.6 (9.1, 15.8)	0.11
**Dietary fiber (g)**	Active disease, ESPEN: Elimination Remission, general population-EFSA: 25 g/day	14.1 (10.5, 19.6)	12.7 (8.4, 15.8)	15.7 (11.5, 21.2)	<0.001	16.1 (12.4, 22.2)	14.9 (11.7, 17.9)	17.7 (13.0, 22.8)	0.01
**Fat (% TEI)**	EFSA: 20–35%	39.6 ± 8.0	40.1 ± 8.8	39.3 ± 7.5	0.45	40.1 ± 7.7	41.6 ± 7.2	39.3 ± 7.9	0.08
**SFA (% TEI)**	EFSA: As low as possible	12.8 (10.6, 14.9)	13.4 (10.8, 15.0)	12.7 (10.5, 14.6)	0.23	12.8 (10.6, 14.8)	13.7 (11.3, 15.0)	12.7 (10.0, 14.6)	0.09
**MUFA (% TEI)**		17.1 (14.3, 20.7)	16.7 (14.5, 21.5)	17.3 (14.3, 20.2)	0.79	17.4 (15.1, 20.8)	18.2 (15.8, 21.5)	17.3 (14.2, 20.5)	0.11
**PUFA (% TEI)**		5.6 (4.6, 7.0)	5.7 (4.6, 6.9)	5.5 (4.6, 7.1)	0.80	5.6 (4.8, 7.2)	5.8 (4.8, 6.9)	5.6 (4.8, 7.3)	0.91
**Trans fatty acids (g)**	EFSA: As low as possible	0.16 (0.03, 0.50)	0.09 (0.03, 0.48)	0.24 (0.03, 0.50)	0.30	0.28 (0.05, 0.58)	0.15 (0.04, 0.56)	0.30 (0.07, 0.59)	0.42

AERs: Adequate energy reporters, BW: Body weight, ESPEN: European Society for Clinical Nutrition and Metabolism, EFSA: European Food Safety Authority, MUFA: monounsaturated fatty acids, PUFA: polyunsaturated fatty acids, SFA: saturated fatty acids, TEI: Total energy intake. Guidelines for protein and fiber intake have been derived from ESPEN [24] and for other macronutrients from EFSA [26]. Desired BW was calculated using body weight corresponding to BMI 20 kg/m^2^ for underweight patients, current body weight for normal weight patients and BMI corresponding to 25 kg/m^2^ for overweight and obese patients. Data for continuous variables are presented as means ± standard deviation (normally distributed variables) or median and 25th–75th percentiles (non-normally distributed variables). * *p* refers to the comparison between active disease and remission in the total sample. ** *p* refers to the comparison between active disease and remission among AERs.

**Table 3:** Food groups consumption, MedDietScore and total score for adherence to healthy diet characteristics for the primary prevention of cardiovascular disease in the total sample and in adequate energy reporters not on a weight loss diet and according to disease activity.

	Greek Dietary Guidelines [38]	Total Sample (*n* = 237, 100%)	Active Disease (*n* = 89)	Remission (*n* = 148)	*p* *	Adequate Energy Reporters (AERs) and Not following a Weight Loss Diet (Ν = 153, 64.6%)	Active Disease (*n* = 53)	Remission (*n* = 100)	*p* **
Full fat dairy (portions/day)	2 portions/day Preference for low-fat	1.21 (0.64, 2.00)	1.14 (0.42, 2.00)	1.21 (0.64, 2.00)	0.70	1.21 (0.63, 2.00)	1.28 (0.42, 2.00)	1.21 (0.64, 2.00)	0.84
Low fat dairy (portions/day)		0.00 (0.00, 0.64)	0.00 (0.00, 0.64)	0.00 (0.00, 0.64)	0.94	0.00 (0.00, 0.21)	0.00 (0.00, 0.07)	0.00 (0.00, 0.64)	0.17
Total dairy products (portions/day)		1.49 (0.89, 2.28)	1.42 (0.71, 2.56)	1.60 (0.96, 2.28)	0.54	1.42 (0.85, 2.28)	1.28 (0.56, 2.56)	1.57 (0.96, 2.28)	0.28
Refined cereals (portions/day)	5–8 portions/day Preference for non-refined	4.98 (2.86, 6.41)	5.03 (2.89, 6.31)	4.98 (2.86, 6.47)	0.84	5.56 (3.31, 6.68)	5.61 (3.70, 6.54)	5.47 (3.05, 6.68)	0.55
Non refined cereals (portions/day)		0.14 (0.02, 0.64)	0.07 (0.00, 0.33)	0.21 (0.02, 0.70)	0.11	0.12 (0.02, 0.42)	0.07 (0.00, 0.28)	0.21 (0.02, 0.61)	0.01
Total cereals (portions/day)		5.33 ± 2.18	5.13 ± 2.21	5.45 ± 2.16	0.29	5.60 ± 2.11	5.55 ± 1.93	5.63 ± 2.21	0.83
Fruits (portions/day)	3 portions/day	1.00 (0.35, 1.71)	0.85 (0.21, 1.35)	1.06 (0.42, 1.92)	0.02	1.06 (0.42, 1.77)	0.92 (0.21, 1.35)	1.31 (0.63, 2.00)	0.01
Fruits and fruit juice ^±^ (portions/day)	Preference for fruits rather than fruit juice	1.35 (0.63, 2.49)	1.20 (0.49, 2.21)	1.46 (0.70, 2.74)	0.01	1.53 (0.70, 2.56)	1.38 (0.46, 2.25)	1.60 (0.84, 2.83)	0.12
Vegetables (portions/day)	4 portions/day	1.00 (0.53, 1.76)	0.89 (0.35, 1.55)	1.11 (0.64, 1.86)	0.02	0.96 (0.49, 1.78)	0.75 (0.35, 1.33)	1.27 (0.61, 2.00)	0.01
Total fruits and vegetables (portions/day)		2.65 (1.45, 4.21)	2.08 (1.30, 3.76)	2.83 (1.72, 4.53)	0.01	2.70 (1.46, 4.47)	2.16 (1.10, 3.72)	2.89 (1.76, 4.78)	0.01
Legumes (portions/week)	3 portions/week	1.26 (0.00, 3.71)	0.00 (0.00, 1.26)	1.26 (0.00, 3.71)	<0.001	1.26 (0.00, 3.71)	0.00 (0.00, 1.26)	1.26 (0.00, 3.71)	<0.001
Fish and fisheries (portions/week)	2–3 portions/week	1.48 (0.50, 2.94)	1.48 (0.50, 2.94)	1.48 (0.50, 2.94)	0.39	1.48 (0.50, 2.94)	1.48 (0.98, 2.94)	1.48 (0.50, 2.94)	0.27
Poultry (portions/week)	1–2 portions/week	1.48 (1.48, 4.48)	1.48 (1.48, 4.48)	1.48 (1.48, 4.48)	0.51	1.48 (1.48, 4.48)	1.48 (1.48, 4.48)	1.48 (1.48, 4.48)	0.54
Red meat (portions/week)	Up to 1 portion/week	3.13 (2.16, 4.62)	3.14 (2.16, 4.56)	3.39 (2.35, 4.62)	0.43	3.64 (2.35, 4.62)	3.64 (2.35, 4.62)	1.30 (3.64, 5.07)	0.65
Cold cuts (portions/week)	Avoidance	1.47 (0.49, 2.73)	1.26 (0.28, 2.73)	1.96 (0.77, 2.73)	0.15	1.96 (0.49, 3.01)	1.26 (0.28, 2.52)	2.24 (0.63, 3.50)	0.20
Total red meat (portions/week)		4.20 (2.80, 5.99)	4.12 (2.66, 5.71)	4.23 (2.94, 6.22)	0.39	4.62 (3.14, 6.36)	4.62 (2.94, 5.94)	4.68 (3.22, 6.50)	0.62
Alcohol (portions/day)	Males: 2 portions/day, Females: 1 portion/day	0.07 (0.00, 0.28)	0.07 (0.00, 0.21)	0.07 (0.00, 0.28)	0.36	0.07 (0.00, 0.28)	0.07 (0.00, 0.21)	0.07 (0.00, 0.28)	0.18
Sweets (portions/week)	Avoidance	4.48 (1.47, 8.40)	3.92 (1.47, 7.91)	4.97 (1.96, 8.96)	0.05	5.46 (2.24, 10.22)	4.97 (1.96, 10.43)	5.95 (2.45, 9.94)	0.78
Soft drinks (portions/week)	Avoidance	0.63 (0.00, 5.88)	1.26 (0.00, 5.88)	0.63 (0.00, 5.88)	0.87	0.28 (0.00, 0.84)	0.28 (0.00, 0.84)	0.18 (0.00, 0.84)	0.21
Coffee and tea (portions/day)		2.00 (0.85, 2.00)	1.07 (0.71, 2.00)	2.00 (1.00, 2.00)	0.11	2.00 (0.85, 2.00)	1.07 (0.71, 2.00)	2.00 (1.00, 2.00)	0.39
Total MedDietScore (0–55)		28.0 ± 5.5	26.8 ± 5.2	28.7 ± 5.5	0.01	28.4 ± 5.5	27.0 ± 5.0	29.1 ± 5.7	0.03
Total score for adherence to European dietary guidelines for CVD prevention (0–11)		5.25 ± 1.36	4.98 ± 1.26	5.42 ± 1.39	0.30	5.35 ± 1.42	4.70 ± 1.29	5.52 ± 1.55	0.01

AERs: Adequate energy reporters, CVD: Cardiovascular disease. Data for continuous variables are presented as means ± standard deviation or median and 25th–75th percentiles and for categorical variables as absolute numbers and relative frequencies. ^±^ Fruit and fruit juices refer to either fresh or condensed/packed fruit juices. * *p* refers to the comparison between active disease and remission in the total sample. ** *p* refers to the comparison between active disease and remission among AERs.

## Data Availability

Not applicable.

## Supplementary Materials

The following supporting information can be downloaded at: https://www.mdpi.com/article/10.3390/nu14245254/s1, Table S1: Food groups and portion sizes according to Greek dietary guidelines. Table S2: Energy and macronutrient intake in low- and adequate-energy reporters with Crohn’s disease. Table S3: Food group consumption, MedDietScore and total score for adherence to healthy diet characteristics for the primary prevention of cardiovascular disease in low- and adequate-energy reporters with Crohn’s disease.
