# Supplementary material for: Assessment of Dietary Adequacy and Quality in a Sample of Patients with Crohn’s Disease
Source: Nutrients. 2022 Dec 9;14(24):5254. doi: 10.3390/nu14245254 (PMC9780994; doi:10.3390/nu14245254)
Supplement: Supplementary file 1 [file nutrients-14-05254-s001.zip › nutrients-2074333-supplementary.pdf]

Supplementary material

Table S1. Food groups and portion sizes according to Greek dietary guidelines.

Food group	Portion size
Vegetables	1 cup raw or cooked (150-200g) green beans, okra, tomato, lettuce, cabbage, greens, cauliflower, tomato juice, broccoli, spinach<br>1 medium cucumber, 2 medium carrots/zucchini
Potato	1 small (120-150g)<br>4-5 pieces of oven-baked potato<br>½ cup fried/mashed
Fruit and fruit juice	1 cup (120-200g): 1 medium apple/ orange/ pear/ nectarine/ grapefruit/ peach/ strawberries/ banana, 1 slice of melon/ watermelon, 2 small tangerines/ apricots/ kiwis, 4 medium plums/ apricots, 30 grapes, 15 cherries, ½ cup freshly squeezed fruit juice (125ml)
Refined grains	30g white bread, 2 white toasts, 30g breakfast cereals, ½ cup (~70-90g) white pasta/ rice, 20g white rusks, 2 white crackers, ½ piece of sesame bagel/pita bread
Non-refined cereals	30g whole wheat bread, 2 whole wheat toasts, 30g whole wheat breakfast cereals, ½ cup (~70-90g) white pasta/ rice/ paddy rice/ groats/ bulgur/ wheat/ corn, 4 tablespoons oats, 20g whole wheat rusk, 2 whole wheat crackers, ½ piece of whole wheat sesame bagel/whole wheat pita bread
Legumes	1 cup of cooked soup or ½ cup of cooked drained (beans, lentils, chickpeas, fava beans, green peas, soya)
Low fat dairy	1 cup of fresh milk 0-2% (250ml), ½ cup of evaporated milk 0-2% (125mL), 1 cup of yogurt 0-2% (200g)
Full fat dairy	1 cup of full fat milk (250ml), ½ cup of full fat evaporated milk (125mL), 1 cup of full fat yogurt (200g)
Cheese	30g feta/gruyere/gouda/edam/yellow cheese/ "mizithra" cheese<br>1 slice of cheese (full fat or low fat)<br>2 tablespoons of soft spread cheese (cottage cheese, cream cheese)
Red meat and products	120-150g pork, beef, lamb, minced meat<br>30g ham, sausage, bacon, salami, bologna etc.
Poultry	120-150g chicken, turkey, minced poultry
Fish and fisheries	150g fish baked/grilled/boiled/fried, seafood
Sweets/Sugar/Honey/Jam	3 cookies, 1 slice of cake (50g), 6 pieces of chocolate (50g), 1 cup of ice cream/rice pudding<br>1 low fat ice cream stick/sorbet, 1 cup of fruit jelly, ½ piece of sesame nougat, ½ cup fruit compote, 1 cereal bar<br>1 teaspoon sugar/honey/jam
Olive oil	Times/day
Coffee	1 cup
Alcohol	330ml beer 4-5%, 125ml wine 11-13%, 40-45ml strong alcohol drinks 40%
Complex foods	Analysis of individual foods from food composition tables

Table S2. Energy and macronutrient intake in low- and adequate-energy reporters with Crohn's disease.

	Low energy reporters, LERs (N=84)	Adequate energy reporters, AERs (N=153)	P*
**Total energy intake (TEI) (kcal/day)**	1479 (1224, 1701)	2295 (1879, 2899)	<0.001
Per kg actual BW (kcal/kg/day)	18.3 (15.1, 21.2)	31.6 (25.8, 39.1)	<0.001
Per kg desired BW (kcal/kg/day)	21.7 (18.6, 24.7)	34.2 (28.0, 41.6)	<0.001
**Protein intake (% TEI)**	19.4±5.6	17.5±4.0	0.01
Per kg actual BW (g/kg/day)	0.88 (0.64, 1.11)	1.36 (1.05, 1.78)	<0.001
Per kg desired BW (g/kg/day)	1.07 (0.81, 1.20)	1.44 (1.17, 1.85)	<0.001
**Carbohydrates (% TEI)**	40.9±8.4	41.4±8.7	0.72
**Sugars (%TEI)**	13.2 (9.6, 17.2)	11.7 (8.8, 15.5)	0.18
**Dietary fiber (g)**	10.8 (7.9, 15.4)	16.1 (12.4, 22.2)	<0.001
**Fat (% TEI)**	38.7±8.8	40.1±7.7	0.25
**SFA (% TEI)**	12.8 (10.4, 14.9)	12.8 (10.6, 14.8)	0.97
**MUFA (% TEI)**	16.3 (13.2, 20.2)	17.4 (15.1, 20.8)	0.09
**PUFA (% TEI)**	5.4 (4.4, 7.0)	5.6 (4.8, 7.2)	0.28
**Trans fatty acids (g)**	0.06 (0.00, 0.35)	0.28 (0.05, 0.58)	<0.001

AERs: Adequate energy reporters, BW: Body weight, LERs: Low energy reporters, MUFA: monounsaturated fatty acids, PUFA: polyunsaturated fatty acids, SFA: saturated fatty acids, TEI: Total energy intake

Desired BW was calculated using body weight corresponding to BMI 20kg/m^2 for underweight patients, current body weight for normal weight patients and BMI corresponding to 25kg/m^2 for overweight and obese patients.

Data for continuous variables are presented as means ± standard deviation (normally distributed variables) or median and 25th – 75th percentiles (non-normally distributed variables).

*p refers to the comparison between LERs and AERs.

Table S3. Food group consumption, MedDietScore and total score for adherence to healthy diet characteristics for the primary prevention of cardiovascular disease in low- and adequate-energy reporters with Crohn's disease.

	**Low energy reporters, LERs (N=84)**	**Adequate energy reporters, AERs (N=153)**	**P***
**Full fat dairy (portions/day)**	1.11 (0.68, 1.93)	1.21 (0.63, 2.00)	0.46
**Low fat dairy (portions/day)**	0.21 (0.00, 0.78)	0.00 (0.00, 0.21)	0.01
**Total dairy products (portions/day)**	1.53 (0.92, 2.25)	1.42 (0.85, 2.28)	0.95
**Refined cereals (portions/day)**	4.09 (2.32, 5.69)	5.56 (3.31, 6.68)	0.01
**Non refined cereals (portions/day)**	0.21 (0.00, 0.90)	0.12 (0.02, 0.42)	0.43
**Total cereals (portions/day)**	4.77 (2.89, 6.30)	5.81 (4.10, 6.89)	0.01
**Fruits (portions/day)**	0.75 (0.28, 1.42)	1.06 (0.42, 1.77)	0.04
**Fruits and fruit juice± (portions/day)**	1.12 (0.49, 2.07)	1.53 (0.70, 2.56)	0.05
**Vegetables (portions/day)**	0.98 (0.58, 1.63)	0.96 (0.49, 1.78)	0.72
**Total fruits and vegetables (portions/day)**	2.27 (1.31, 3.66)	2.70 (1.46, 4.47)	0.11
**Legumes (portions/week)**	1.26 (0.00, 1.26)	1.26 (0.00, 3.71)	0.14
**Fish and fisheries (portions/week)**	1.48 (0.50, 2.94)	1.48 (0.50, 2.94)	0.77
**Poultry (portions/week)**	1.48 (1.48, 4.48)	1.48 (1.48, 4.48)	0.25
**Red meat (portions/week)**	3.14 (2.02, 4.12)	3.64 (2.35, 4.62)	0.04
**Cold cuts (portions/week)**	1.26 (0.28, 2.73)	1.96 (0.49, 3.01)	0.19
**Total red meat (portions/week)**	3.44 (2.46, 5.38)	4.62 (3.14, 6.36)	0.01
**Alcohol (portions/day)**	0.04 (0.00, 0.25)	0.07 (0.00, 0.28)	0.09
**Sweets (portions/week)**	3.43 (1.47, 6.44)	5.46 (2.24, 10.22)	0.01
**Soft drinks (portions/week)**	0.63 (0.00, 1.96)	0.28 (0.00, 0.84)	0.19
**Coffee and tea (portions/day)**	1.82 (1.00, 2.07)	2.00 (0.85, 2.00)	0.67
**Total MedDietScore (0-55)**	27.1±5.4	28.4±5.5	0.09
**Total score for adherence to European dietary guidelines for CVD prevention (0-11)**	4.90±1.20	5.35±1.42	0.07

AERs: Adequate energy reporters, CVD: Cardiovascular disease, LERs: Low energy reporters

Data for continuous variables are presented as means ± standard deviation or median and 25th – 75th percentiles and for categorical variables as absolute numbers and relative frequencies.

±Fruit and fruit juices refer to either fresh or condensed/packed fruit juices.

*p refers to the comparison between LERs and AERs.
